# Quality of Life of Families with Children Presenting Congenital Heart Disease:Longitudinal Study Protocol

**DOI:** 10.3390/healthcare10071273

**Published:** 2022-07-09

**Authors:** Silvio Simeone, Teresa Rea, Nicol Platone, Assunta Guillari, Aniello Lanzuise, Nadia Assanta, Paola Da Valle, Stefania Baratta, Gianluca Pucciarelli

**Affiliations:** 1 Clinical and Experimental Medicine Department, “Magna Graecia” University, 88100 Catanzaro, Italy; silviocecilia@libero.it; 2 Public Health Department, Federico II University Hospital, 80131 Naples, Italy; teresa.rea@unina.it; 3 Gaetano Pasquinucci Heart Hospital, 54100 Massa, Italy; nicole.platone@gmail.com (N.P.); assanta@ftgm.it (N.A.); davalle@ftgm.it (P.D.V.); stefania.baratta@ftgm.it (S.B.); 4 Department of Public Health, University Federico II of Naples, 80145 Napoli, Italy; 5 S. Maria della Pietà Hospital-Camillians, 80026 Casoria, Italy; alanzuise@gmail.com; 6 Department of Biomedicine and Prevenion, University of Rome Tor Vergata, 00133 Rome, Italy; g.pucciarelli81@gmail.com

**Keywords:** congenital heart disease, protocol, interdependence, QoL, family

## Abstract

BACKGROUND: Advances in medicine have caused a notable increase in the survival rates of children born with congenital heart disease, even in the most complicated cases, almost mitigating the disease’s pathology from lethal to chronic. The quality of life perceived by such children is influenced by the perceptions of their parents. However, the international literature has rarely considered the entire family nucleus. AIMS: This study aims to study the temporal trend of quality of life of families with children with congenital heart disease, particularly with respect to parents following a child’s hospitalization for an invasive procedure. DESIGN: A longitudinal study. METHOD: A sample of families (that is, those including a child with congenital heart disease and their parents) will be enrolled following the patient’s discharge from the hospital and examined every 3 months for 1 year. The study’s adopted hypothesis is that there is an interdependence between the subjects of the study that is capable of influencing individual perceptions of quality of life. RESULTS: This study will attempt to identify variables (and their temporal trend) that can be attributed to the family unit and—together with physical and clinical variables—that may influence the quality of life of children with congenital heart disease. CONCLUSION: Examining family quality of life with the longitudinal method will allow us to identify the predictors and interdependence of this factor with respect to children and their parents. This will help to correct and elaborate upon care guidelines, providing better assistance to patients and their caregivers.

## 1. Background

Congenital heart defects (CHDs) represent a broad spectrum of defects caused by the abnormal development of the heart during the fetal stage [1]. The current prevalence of CHD is 8 in 1000 live births [2,3]. Around 150 million live births are recorded every year around the world, of which 1.35 million are affected by congenital heart disease [1]. CHD is, consequently, one of the most common birth malformations, diagnosed in about 400,000 babies born each year [4]. Of these, about 25% have complex CHDs with high mortality rates [5]. However, thanks to improvements in medical procedures over the last 30 years, today about 85% of children with CHD reach adulthood [6].

Additionally, given that CHDs represent different types of defects, which can be grouped into cyanotic and non-cyanotic—or, simply, moderate and complex [7]—even after a successful operation, many children will need to undergo follow-up surgeries and/or diagnostic curative procedures [8]. Children who survive such heart surgery appear to have different neurodevelopmental risks compared to those experienced by their peers [9]. Furthermore, evidence has emerged of delays in growth and other exacerbated symptoms, which may impose physical restrictions on the child and affect their quality of life (QoL) [10]. As mentioned, improvements in care have paved the way for a significant increase in the survival rates of these children, almost transforming their disease’s pathology from lethal to chronic [11,12].

The international literature emphasizes the need for children with CHD to have a smooth transition into adulthood in order to optimize their QoL [13]. When children with heart disease return home after heart surgery or an invasive procedure, the parents, who are often their primary caregivers, experience an increase in their workload [14]. Additionally, they also undergo a consequent increase in their stress levels and depression [15]. Furthermore, as in other populations, caregiving with respect to pediatric subjects with CHD can lead to social isolation, which can heighten the self-perception of the caregivers’ psychological disorders [16]. This increased workload, and the feelings that may arise within the parental dyad after a child’s return, can affect the family environment and the child him or herself [14,17]. The children of parents suffering from PTSD have an increased risk of developing sleep and eating disorders, leading to a higher number of hospital admissions [16]. These parental and family factors may have a greater effect on CHD outcomes than the type of heart defect or the course of surgical palliation [18].

Although the relationship between a child’s behavior, development, chronic condition, vulnerability and parental stress has been documented [19,20], most studies have only focused on the main physical and psychosocial consequences of CHD, with little attention paid to the relationship between the child’s condition and the QoL of the parents [21,22,23]. Within the family unit (i.e., consisting of parents and a child with CHD), negative interactions between members, such as reduced interactions with other family members, restrictions on social life, increased distress or subjective tension, have been reported [24,25]. Interestingly, the current scientific evidence regarding the correlation between the QoL of children with CHD and the family focuses almost exclusively on the mother [26]. This prevents the family unit from being considered correctly and shows a major bias in educational interventions [23]. There is a lack of data on how the QoL of children impacts and/or is influenced by that of the family in the long run [27]. Given this, more longitudinal studies that include entire families (i.e., the mother, father and child with CHD) and that investigate interactions between their qualities of life [23] are strongly recommended. Furthermore, although the international literature attributes a key role to the caregivers of the children with CHD in guaranteeing their out-of-hospital care, their QoL in Italy has been poorly studied. We, therefore, do not know if there is an interaction between child QoL and parent QoL. As a matter of fact, to date, little is known about the QoL of children with CHD who reach adulthood: those who have grown-up with congenital heart disease (GUCH) [13,28].

## 2. Aims

This study has the following objectives:

(a) To study the QoL of children and their informal caregivers after the former have undergone cardiac surgery and/or palliative/curative study procedures linked to CHD upon discharge from the hospital;
(b) To study, with longitudinal drawing, the progress of the QoL of children and their parents in the first 6months after cardiac surgery and/or study/palliative/curative procedures linked to CHD;
(c) To identify the predictors of the QoL of children and parents in order to direct the correct development of guidelines to improve nursing care for patients and caregivers;
(d) To analyze the interdependence between the child–parent dyad in the population with CHD.

## 3. Hypothesis

The QoL of children influences and is affected by the QoL of the parental dyad. There is an interdependence between the study subjects capable of influencing individual perceptions of QoL.

Over time, the perception of QoL varies based on individual perceptions of the family’s QoL.

### 3.1. Design

This study adopts a longitudinal descriptive design with data collection planned at 0, 3 and 6 months after the children are discharged from operational units.

### 3.2. Sampling

A convenience sample consisting of 350 children with CHD and their parents is expected to be enrolled.

### 3.3. Size Sampling

The aforementioned value was calculated with a confidence level of 95% and a confidence interval of 5%,starting from the real figure of 400,000 new births with CHD in the world (8/1000) per year [29], with the total birth value in Italy reaching up to 458,151 [30]. Furthermore, a similar sample value was used for the psychometric validation of PedsQL scales aimed at assessing the QoL of similar subjects (children with CHD and their parents) in Spanish [31].

### 3.4. Inclusion Criteria for Patients

(a) Children with CHD/subjects with grown-up CHD (GUCH) undergoing cardiac surgery and/or hemodynamic study/palliative/curative procedures after being discharged from hospital units and whose parents have given consent to take part in the study;
(b) Children over 5 years of age.

### 3.5. Exclusion Criteria for Patients

(a) Severe neurological deficits following intervention;
(b) Severe organ deficiency.

### 3.6. Inclusion Criteria for Caregivers

(a) Being the parents of children with CHD or the informal caregiver of a person with CHD. For the purposes of this study, caregivers are defined as parents who care for or take responsibility for a child with CHD;
(b) Consent to take part in the study is provided;
(c) Being legally married, cohabiting, or otherwise living together in the same place.

### 3.7. Exclusion Criteria for Caregivers

- Not legally married, cohabiting, or, in any case, cohabiting in the same place;
- Willingness to withdraw from the study (even from only one member);
- Child’s death.

## 4. Data Collection and Setting

### 4.1. Setting

The study will be conducted in the homes of the patients, electronically, except for time “0” or in relation to hospital discharge. All enrolled subjects will be asked to simply fill in validated questionnaires aimed at assessing the QoL trends and identifying possible predictors of their specific trajectories. The questionnaires will be electronically sent to them and will only be user-accessible through the appropriate link.

### 4.2. Variables and Data Collection Tools

Valid and reliable research tools, administered to both children and caregivers (parents), will be used to collect the data. For specifics, see Table 1.

### 4.3. For Patients

(*) Sociodemographic questionnaire: This tool will be useful for collecting data regarding age, sex, marital status, occupation, education and other details.

(*) Clinical card: This will be used to collect clinical data such as the date and type of intervention and the severity level and the type of CHD.

(*) Pediatric Quality of Life Inventory 3.0 Cardiac Module [32,33]: Scales derived from the Pediatric Quality of Life Inventory 4.0 have been developed for the specific assessment of the QoL of children with CHD and their parents. This is formed by five domains related to symptoms (seven items), perceived physical appearance (three items), anxiety treatment (four items), cognitive problems (five items) and communication (three items), validated for children (8–18 years) and in a proxy version for the parents (2–18 years) [32]. Additionally, for this scale, there are different formats for children, divided into three age groups (5–7 years, 8–12 years and 13–18 years) and for the parents of such children. A five-point Likert-type scale is used for children aged 8–18 and their parents, and a three-point scale is used for younger children for ease of use. In our study, the 2–4-year-old population is excluded. Compilation times are extremely short (about 5 min in total for both parents and children) [33].

(*) For subjects over 18 years old: The WHOQOL-BREF [34] tool consists of 26 questions with answers structured on a five-point Likert-type scale designed to assess perceived QoL. A self-administered questionnaire focuses on the respondent’s perception of the 2 weeks prior to the survey. The tool’s first two questions assess the general QoL. The WHOQOL-BREF also evaluates four dimensions of QoL: physical, psychological, social and environmental. For the purposes of the categorical assessment of QoL, the WHOQOL-BREF has a cutoff point of ≥60 points for acceptable levels. Closer to 100 points, the highest population QoL levels are studied.

### 4.4. For Caregivers

(*) Sociodemographic questionnaire: This aims to collect data related to the caregiver, such as age, sex, education, profession, marital status, degree of kinship and coexistence with the patient and hours of assistance provided to the patient.

(*) Pediatric Quality of Life Inventory 3.0 Cardiac Module [32,33]: Scales derived from the Pediatric Quality of Life Inventory 4.0 have been developed for the specific assessment of the QoL of children with CHD and their parents. It is composed of five domains related to symptoms (seven items), perceived physical appearance (three items), treatment anxiety (four items), cognitive problems (five items) and communication (three items) validated for both children (8–18 years) and in a proxy version for the parents (2–18 years) [32]. Furthermore, for this scale, there are different formats for children, divided into three age groups (5–7 years, 8–12 years and 13–18 years) and for the parents of such children. A five-point Likert-type scale is used for children aged 8–18 years and their parents, and a three-point Likert-type scale is used for younger children for ease of use. In our study, the 2–4-year-old population is excluded. Compilation times are extremely short (about 5 min in total for both parents and children) [33].

(*) PHQ9 [35]: A subscale composed of nine items within the PHQ. This scale is used, even in self-administration, for the monitoring and determination of depression. The answers for each question are based on a Likert scale, and the total score has a range between 0 and 27.

(*) Hospital Anxiety and Depression Scale [36]: This is a scale composed of 14 items grouped into two subscales that measure anxiety and depression. Each subscale can have a score from 0 to 21: A high score corresponds to high levels of anxiety and depression. This is meant to assess the levels of anxiety and depression in the parents of children undergoing cardiac surgery [25]; this scale is used to measure these outcomes even after hospitalization and discharge [37].

(*) For caregivers of patients over 18 years of age: The WHOQOL-BREF [34] tool consists of 26 questions with structured answers on a five-point Likert-type scale designed to assess perceived QoL. A self-administered questionnaire focuses on the respondent’s perception of the 2 weeks prior to the survey. The tool’s first two questions assess the general QoL. The WHOQOL-BREF also evaluates four dimensions of the QoL: physical, psychological, social and environmental. For the purpose of the categorical assessment of QoL, the WHOQOL-BREF has a cutoff point of ≥60 points for acceptable levels. Closer to 100 points, the highest population QoL levels are studied.

(*) Caregiver Burden Inventory [38]: This is a multidimensional tool, initially developed for the caregivers of Alzheimer’s patients, and it evaluates care burden. It is a self-report tool and uses a five-point Likert scale with a scale ranging from “Not at all” to “Very” for answers. It is divided into five sections: objective load, psychological load, physical load, social load and emotional load. It has been used in several studies on Italian caregivers [39,40,41] as well as on the caregivers of pediatric patients [42].

(*) Preparedness for Caregiving Scale [43]: This is a scale that measures the caregiver’s preparation in coping with the task of caregiving. The areas investigated by the scale concern the preparation for physical assistance, emotional support and the ability to find support services and cope with the stress of care giving. It is made up of eight items using a five-point Likert scale. In addition, there is also an open question that asks the caregiver to identify additional areas in which they would like to be prepared. The possible score ranges from 0 to 40: A high score means the caregiver is better prepared for caregiving.

### 4.5. Data Analysis

Descriptive statistics (mean, standard deviation, median, frequency) will be used to summarize sociodemographic data, clinical data and scale scores.

The Pearson and Spearman correlations will be used to identify which variables will be correlated with the QoL of the dyad. In addition, via multilevel analysis, linear regression will be used to identify the predictors of QoL for the child–family dyad within the CHD population. The actor–partner interdependence model (APIM) will be used to analyze data drawn from the dyads so that we can observe how one aspect affects another. In APIM, the actor effect is the influence of a person’s emotions on themselves (for example, the effect of depression on their QoL), while the partner effect is the impact of the person’s emotions on their partner (for example, the effect of the person’s depression on the partner’s QoL). The APIM will be used as an analytical dyadic procedure to verify how some CHD variables (for example, multigroup analysis) can be used to test the effect of moderators (for example, social support for dyads with high and low QoL).

### 4.6. Ethical Considerations

This study fully complies with the Helsinki Declaration. The protocol has been approved by the Tuscany Northwest Area Committee.

The parents of children scheduled for hospitalization at FTGM will receive an email about the study 7 days before their admission. On the day of prehospitalization, they will be asked if they clearly understand the information. If they show interest in the study, the purposes and methods of carrying it out will be explained again. Upon signing the informed consent form, they will provide their email addresses, to which a link will be sent within 48 h of hospital discharge. This will help them complete their participation in the study. Subsequently, the patients and caregivers will be contacted after 3 months to readminister the aforementioned research tools, except for the sociodemographic questionnaire for family members and children and the patient’s clinical record.

### 4.7. Validity and Reliability/Rigor

In this study, tools that have already been tested for validity and reliability will be used. The validity of the contents of those tools developed by the research team, such as the sociodemographic questionnaires, was assessed by a group of experts. We will also test the interrater reliability of these tools.

## 5. Discussion

Health-related QoL is conceptualized as a holistic and multifaceted construct [44] which includes both physical and psychosocial functioning and can be assessed by general or disease-specific QoL measures [10].

The international literature argues that patients’ perspectives and, therefore, their health and perceived QoL should be regularly assessed by healthcare professionals in order to receive a correct and complete view of their patients’ statuses [45].

While QoL has been studied before in pediatric patients with CHD undergoing surgery [7], there is a lack of data on how child QoL affects and/or is influenced in the long run by family QoL [27]. Longitudinal studies that include the entire family (i.e., the mother, father and child with CHD) and that investigate the interactions between their qualities of life are strongly recommended [23].

It is known that the surgical complexity of the interventions these patients undergo has little influence on their long-term QoL [46]. As a matter of fact, it has been shown that surgical factors, ICU admissions, demographic variables and the use of medical care explain only a modest amount (about 25%) of variation in QoL scores within this specific long-term population. In fact, while the complexity of CHD and surgery is decisive with respect to perceived QoL in the immediate postoperative period, congenital heart diseases with similar severity can have different outcomes regarding long-term perceived QoL scores. Based on these data, other factors contribute to QoL variability [47].

To better understand a complex construct such as QoL, it is essential to investigate the perspectives of the patient and both parents. Clarifying the relative impact of the predictors of QoL is essential to targeting clinical interventions as well as to guiding significant and innovative research [44].

## 6. Conclusions

The QoL of children with CHD after surgery is not only influenced by “clinical” and “physical” variables but also by variables attributable to the family unit. All family members influence and are influenced by the QoL of such children. Studying family QoL longitudinally will allow us to identify the predictors and interdependence of this factor with respect to children and their parents, helping us to direct the correct elaboration of care guidelines, which will improve nursing care for both patients and caregivers.

## Figures and Tables

**Table 1:** Variables and Instruments that Will Be Used for Data Collection: Individual Measures.

Operationalized as	Measured by	Children	Parents (Mother)	Parents (Father)	Measured at (Months)
Sociodemographic	Sociodemographic questionnaire	X	X	X	0
Clinic Status	Medical record	X			0
Quality of Life (5–18 years)	Pediatric Quality of Life Inventory 3.0	X	X	X	0-3-6
Quality of Life (over 18 years)	WHOQOL-BREF	X	X	X	0-3-6
Depressive State	PHQ9		X	X	0-3-6
Anxiety	HADS		X	X	0-3-6
Caregiver Burden	Caregiver Burden Inventory		X	X	0-3-6
Preparedness for Care giving	Preparedness for Care giving Scale		X	X	0-3-6

## Data Availability

Not applicable.
